# 

**Published:** 1858-01-01

**Authors:** 


### THE JOURNAL

OF

### PSYCHOLOGICAL MEDICINE

AND

### MENTAL PATHOLOGY.

EDITED BY

FORBES WINSLOW, M.D., D.C.L. Oxon.

VOL. XI.

Gen'l's Office
LIBRARY.
22055
Washington, D.C.

LONDON:
JOHN CHURCHILL, NEW BURLINGTON STREET.
MDCCCLVIII.

W
1
JO 857 N

LONDON:
SAVILL AND EDWARDS, PRINTERS, CHANDOS STREET,
COVENT GARDEN.

